# On triangle inequalities of correlation-based distances for gene expression profiles

**DOI:** 10.1186/s12859-023-05161-y

**Published:** 2023-02-08

**Authors:** Jiaxing Chen, Yen Kaow Ng, Lu Lin, Xianglilan Zhang, Shuaicheng Li

**Affiliations:** 1 Department of Computer Science, City University of Hong Kong, Hong Kong, China; 2 Department of Computer Science, Beijing Normal University - Hong Kong Baptist University United International College, Zhuhai, People’s Republic of China; 3 State Key Laboratory of Pathogen and Biosecurity, Beijing Institute of Microbiology and Epidemiology, Beijing, 100071 People’s Republic of China

**Keywords:** Correlation, Distance, Triangle inequality, Clustering, Gene expression analysis, Single cell

## Abstract

**Background:**

Distance functions are fundamental for evaluating the differences between gene expression profiles. Such a function would output a low value if the profiles are strongly correlated—either negatively or positively—and vice versa. One popular distance function is the absolute correlation distance, $d_a=1-|\rho |$, where $\rho$ is similarity measure, such as Pearson or Spearman correlation. However, the absolute correlation distance fails to fulfill the triangle inequality, which would have guaranteed better performance at vector quantization, allowed fast data localization, as well as accelerated data clustering.

**Results:**

In this work, we propose $d_r=\sqrt{1-|\rho |}$ as an alternative. We prove that $d_r$ satisfies the triangle inequality when $\rho$ represents Pearson correlation, Spearman correlation, or Cosine similarity. We show $d_r$ to be better than $d_s=\sqrt{1-\rho ^2}$, another variant of $d_a$ that satisfies the triangle inequality, both analytically as well as experimentally. We empirically compared $d_r$ with $d_a$ in gene clustering and sample clustering experiment by real-world biological data. The two distances performed similarly in both gene clustering and sample clustering in hierarchical clustering and PAM (partitioning around medoids) clustering. However, $d_r$ demonstrated more robust clustering. According to the bootstrap experiment, $d_r$ generated more robust sample pair partition more frequently (*P*-value $<0.05$). The statistics on the time a class “dissolved” also support the advantage of $d_r$ in robustness.

**Conclusion:**

$d_r$, as a variant of absolute correlation distance, satisfies the triangle inequality and is capable for more robust clustering.

**Supplementary Information:**

The online version contains supplementary material available at 10.1186/s12859-023-05161-y.

## Introduction

In biological data analysis, we regularly evaluate the similarity between two gene expression profiles. For example, when identifying gene expression patterns across different conditions, when clustering genes of similar functions [1, 2], when detecting the gene temporal profile of relevant functional categories by time-series data clustering [3], when measuring similarity between genes in microbial community [4], and when inferring gene regulatory networks [5].

Several distance functions are commonly adopted to evaluate the similarity—the most prominent one being the absolute correlation distance. The function regards positive correlation and negative correlation equally, giving a value close to zero to highly correlated profiles (whether positively or negatively correlated), and a value of one to uncorrelated profiles. More precisely, the absolute correlation distance is defined as $d_a=1-|\rho |$, where $\rho$ can be Pearson correlation, Spearman correlation, uncentered Pearson correlation (which is equivalent to Cosine similarity), or Kendall’s correlation. Profiles which are perfectly correlated have $\rho =1$ or $\rho =-1$, and hence resulting in $d_a=0$; profiles which are uncorrelated have $\rho =0$, hence resulting in $d_a=1$. The absolute correlation distance is widely used, for example, in measuring the co-expression similarity between the profiles of genes in WGCNA [6], clustering of gene expression [7], and in defining the abundance similarity between OTUs in microbiome area [4]. However, despite its widespread usage, it has been noted that most variants of the measure, except for the absolute Kendall’s correlation, suffer from the drawback of dissatisfying the triangle inequality [3, 8]. For example, consider the vectors $x=(6,4,9), y=(6,9,5), z=(3,10,7)$. Using Pearson correlation as $\rho$, $d_a(x,y)=0.077, d_a(y,z)=0.339, d_a(x,z)=0.679$, resulting in $d_a(x,y)+d_a(y,z) < d_a(x,z)$, thus the triangle inequality fails.

**Fig. 1:**
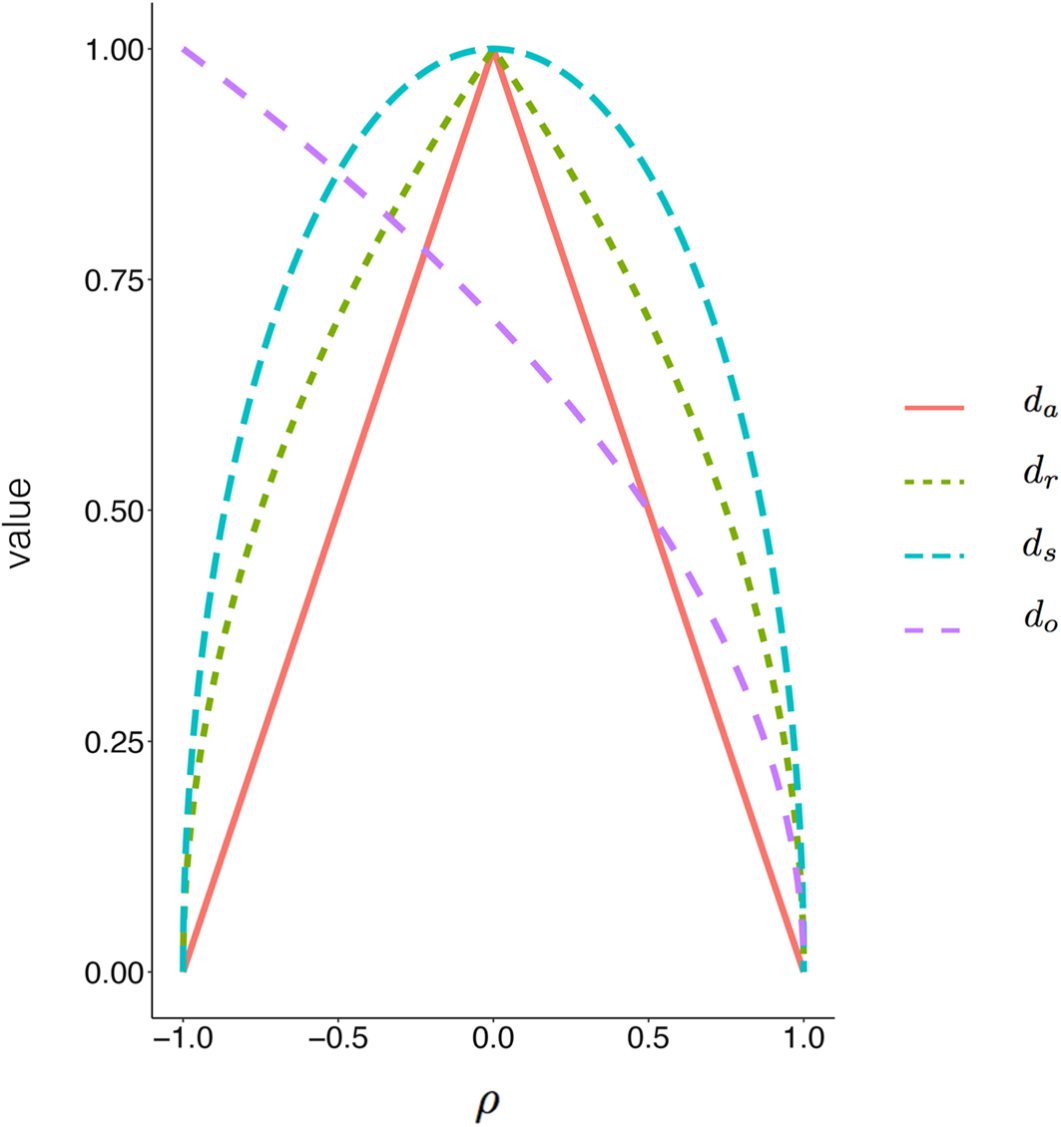
Curve of $d_a$, $d_r$, $d_s$ and $d_o$. $\rho$ (*x*-axis) is plotted against the value of $d_a$, $d_r$, $d_s$ and $d_o$ (*y*-axis). Values are calculated according to their respective equation. The curve of $d_r$ fits $d_a$ better than the curve of $d_s$

A distance measure *d* which (1) satisfies the triangle inequality and (2) has $d(x, y) = 0$ when $x = y$, is called a *metric* [9, 10]. Researchers have observed that the performance of vector quantization improves when the measure used is a metric [11]. A measure which fulfills the triangle inequality would allow faster data localization as well as accelerated data clustering [9, 12]. Many clustering algorithms, such as k-means [13] and DBSCAN [14], can exploit the triangle inequality to achieve better performance. For instance, a distance calculation can be skipped as soon as it is found to exceed the lower or upper bounds estimated through the triangle inequality [13]. The same strategy cannot be applied on distance measures that violate the triangle inequality without compromising the quality of the clustering [9].

Variants of the absolute correlation distance are not the only distance measures used in gene expression analysis that violate the triangle inequality. Prasad et al. [15] compiled a list of distance measures for the analysis of gene expression profiles. Many of the measures in the list failed to fulfill triangle inequalities. These include the Harmonically summed Euclidean distance, Bray-Curtis distance, Pearson correlation distance, absolute Pearson correlation distance, uncentered Pearson correlation distance, absolute uncentered Pearson correlation distance, Pearson linear dissimilarity, Spearman correlation distance, absolute Spearman rank correlation, and the Cosine distance. Recently, Van Dongen et al. [16] proposed two variants of the absolute correlation distance, namely $d_o=\sqrt{\frac{1}{2}(1-\rho )}$ and $d_s=\sqrt{(1-\rho ^2)}$, both of which are metrics. The first variant, $d_o$, demonstrates behavior that is markedly different from $d_a$ (see Fig. 1). When $\rho =-1$, $d_o$ gives a value of 1, whereas $d_a=0$. This excludes it from applications that require absolute distance measures. The other variant, $d_s$, defined as $\sqrt{1-\rho ^2}$, is better as a replacement for $d_a$. However, the performance of $d_s$, especially in clustering biological data, has not been evaluated in [16].

**Fig. 2:**
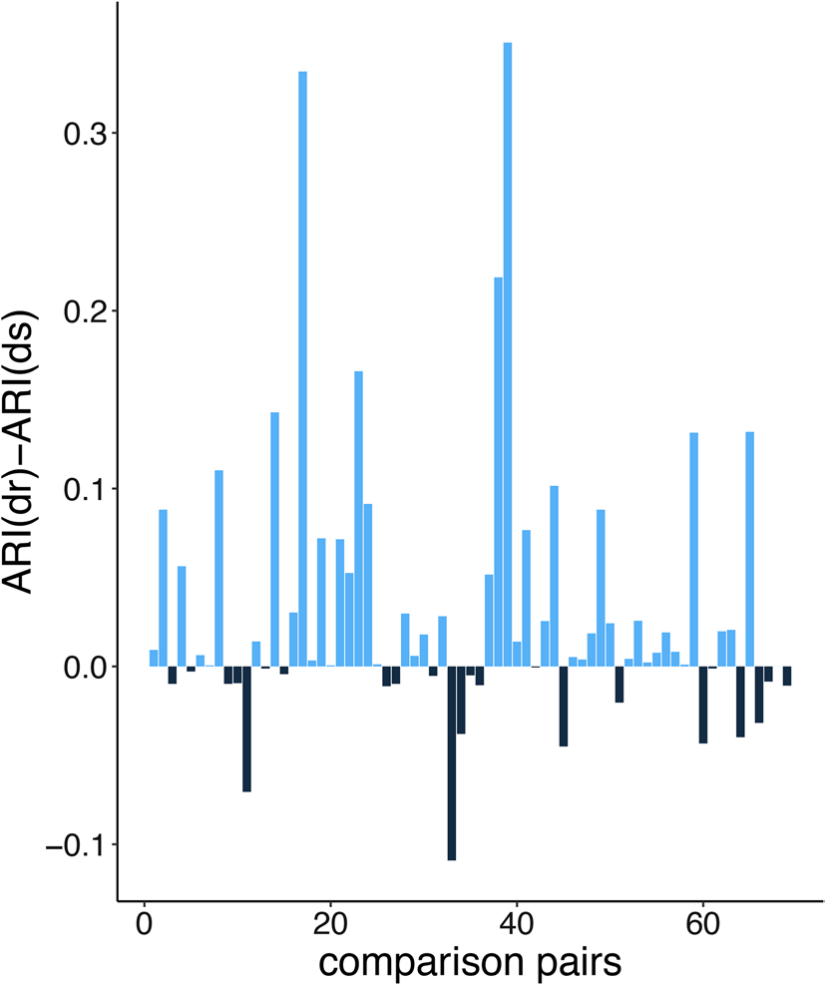
ARI differences of $d_r$ and $d_s$ in sample clustering with hierarchical clustering. ARI difference of $d_r$ and $d_s$ with those pairs ARI are different in $d_r$ and $d_s$. Comparison pairs include datasets in hierarchical clustering when $\rho$ refers to Pearson correlation, Spearman correlation and uncentered Pearson correlation. Each bar refers to one pair. *y*-axis refers to ARI acquired by $d_r$ minus that of $d_s$

In this work, we propose an alternative, $d_r$, to the absolute correlation distance, defined as $d_r=\sqrt{1-|\rho |}$. We show that the function is a metric when $\rho$ is Pearson correlation, Spearman correlation, or uncentered Pearson correlation (or Cosine similarity). Analytically, $d_r$ demonstrates behavior that is more consistent with $d_a$ than $d_s$. We hence expect the traditionally good performance of $d_a$ in clustering tasks to carry over to $d_r$.

There are previous works that discuss the gene expression distance in clustering. Priness et al. [17] applied mutual information distance to the gene expression clustering problem. Zapala et al. [18] proposed a multivariate regression analysis of distance matrices. Jaskowiak et al. [19] evaluated 15 distances in the gene expression clustering problem. None of them discussed the triangle inequality in the distance measurement. Our work focuses on correlation-based distance and propose a distance function $d_r$, a correlation-based distance, to satisfy the triangle inequality.

We compared the performance of $d_r$ to $d_a$ (as well as $d_s$) in biological data clustering. The clustering method includes hierarchical clustering and PAM (partitioning around medoids) [20]. For $\rho$ we used Pearson correlation, Spearman correlation, and Cosine similarity. As data, we used 16 normalized time-series datasets, cancer sample clustering in 35 expression datasets, and cell clustering in seven single cell datasets. Performances for the sample clustering tests were evaluated with the adjusted Rand index (ARI) [21], while those for the gene clustering tests were evaluated with functional analysis.

**Fig. 3:**
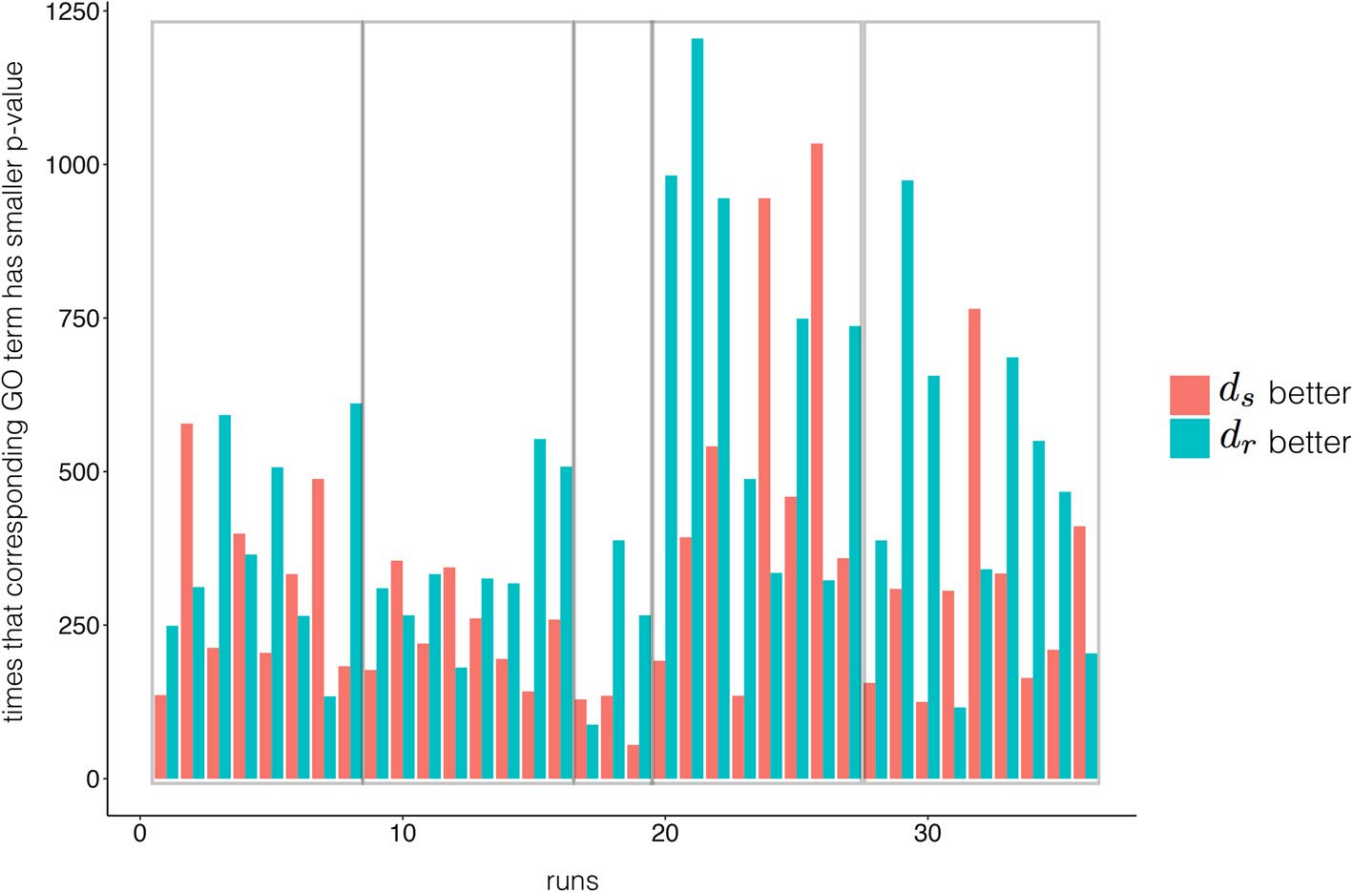
Numbers of GO terms obtained by $d_r$ and $d_s$ in gene clustering with significant difference. Numbers of GO terms is obtained by $d_r$ and $d_s$ respectively. The figure includes pairs with significant difference in *P*-values for corresponding GO terms in $d_r$ and $d_s$. Each bar implies one run (certain distance measure and clustering method in one data set), and the adjacent red and green bar refer to one comparison (only the distance measure is different). Grey rectangular from left to right include comparison pairs in Pearson correlation, Spearaman correlation, uncentered Pearson correlation with hierarchical clustering, and Pearson correlation, Spearaman correlation with PAM. Uncentered Pearson correlation with PAM have no GO terms that have significant difference. *y*-axis refers to the number of times that the GO term of $d_s$ has smaller *P*-value than that of $d_r$ (red bar), and vice versa

Our results show $d_r$ to perform better at clustering than both $d_a$ and $d_s$. $d_r$-based clustering outperformed $d_s$ in sample clustering when the hierarchical clustering is applied and $\rho$ is Pearson correlation and uncentered Pearson correlation (*P*-value$<0.1$, Wilcoxon test). $d_r$-based clustering also generates significantly more meaningful gene clusters than $d_s$ (*P*-value$<0.05$, Wilcoxon test), when considering the corresponding GO-term (Gene Ontology term) with significant difference in *P*-value. $d_r$ and $d_a$ have comparable performances in real sample clustering, while $d_r$ slightly outperforms $d_a$ in gene clustering (*P*-value$<0.1$, Wilcoxon test, besides PAM with uncentered Pearson correlation). Moreover, the clustering performed with $d_r$ is more robust than that with $d_a$. When tested with multiple bootstrap tests, $d_r$ led to more robust clusters than $d_a$ in both hierarchical clustering, when considering internal nodes, and PAM clustering when any of the correlations is used as $\rho$. For PAM clustering with Pearson correlation used as $\rho$, in more than 34 datasets (35 in total), $d_r$ generated significantly (*P*-value $<0.05$) more robust sample pair partition than $d_a$. Similar results were obtained when $\rho$ is Spearman correlation and Cosine similarity. The robustness of $d_r$ is also supported by statistics on the time a class “dissolved” [22]. That makes $d_r$ a good replacement of $d_a$, for $d_r$ has the comparable accuracy and higher robustness, moreover, the possibility to speed up clustering given it fulfills the triangle inequality.

## Method

### Proof for the triangle inequality of $d_r$

The original absolute correlation distance $d_a=1-|\rho |$ dissatisfies the triangle inequality. We propose a new measure $d_r$, as

$$\begin{aligned} d_r (X, Y)=\root \of {1-|\rho _{XY}|} \end{aligned}$$

where *X* and *Y* are expression profiles, and $\rho$ can be any one of Pearson correlation, Spearman correlation, or uncentered Pearson correlation. We claim that $d_r$ satisfies the triangle inequality, when $\rho$ is Pearson correlation, Spearman correlation, or the uncentered Pearson correlation.

**Fig. 4:**
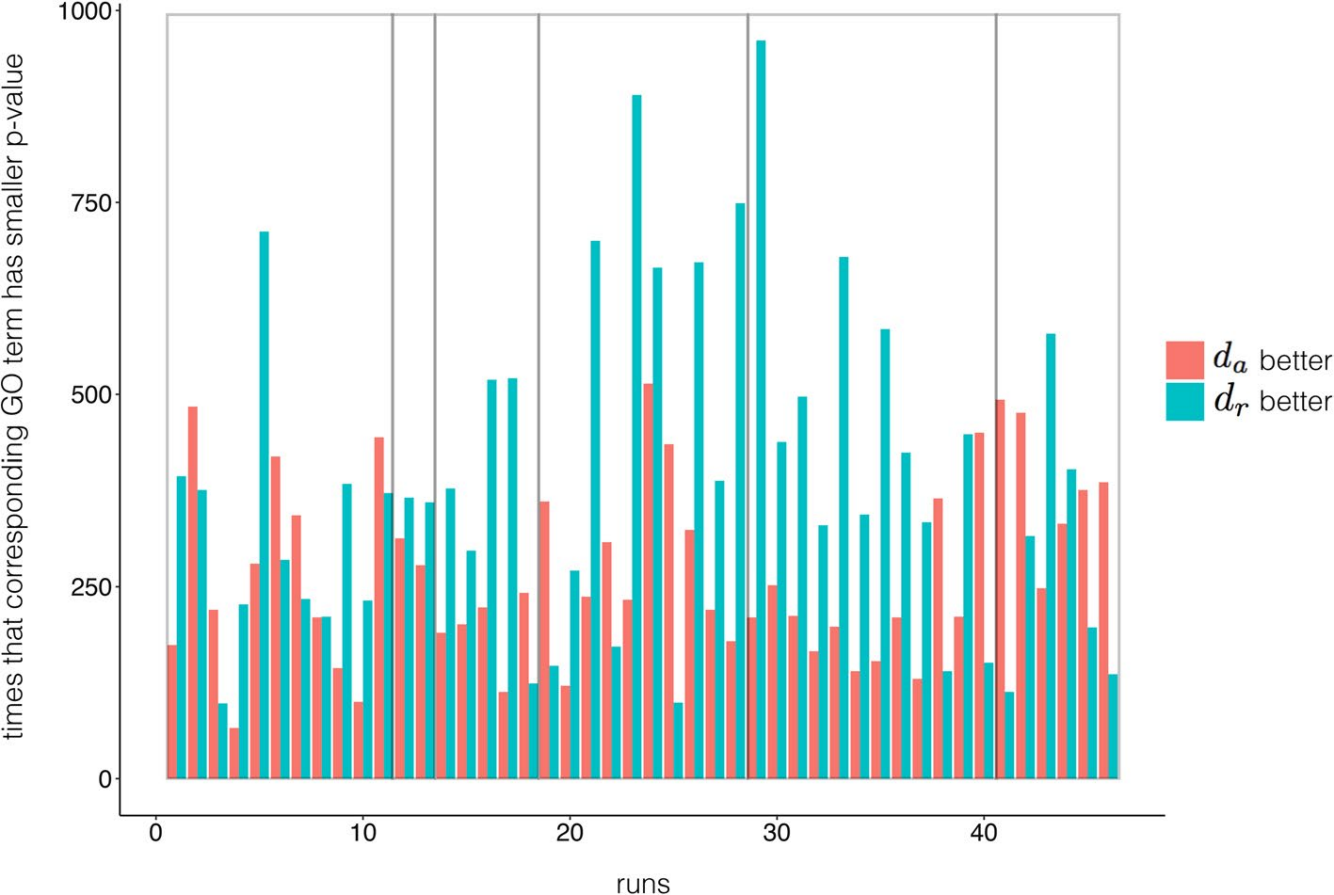
Numbers of GO terms obtained by $d_r$ and $d_a$ in gene clustering with significant difference. Numbers of GO terms is obtained by $d_r$ and $d_a$ respectively. The figure includes pairs with significant difference in *P*-values for corresponding GO terms in $d_r$ and $d_a$. Each bar implies one run (certain distance measure and clustering method in one data set), and the adjacent red and green bar refer to one comparison (only the distance measure is different). Grey rectangular from left to right include comparison pairs in Pearson correlation, Speraman correlation, uncentered Pearson correlation with hierarchical clustering, and the three with PAM. *y*-axis refers to the number of times that the GO term of $d_a$ has smaller *P*-value than that of $d_r$ (red bar), and vice versa

First consider the case where $\rho$ is the Pearson correlation. By the triangle inequality of distance in *n*-dimensional Euclidean space, $d_r(X,Z) \le d_r(X,Y)+d_r(Y,Z)$, where $X=(x_1, x_2,..., x_n)$, $Y=(y_1, y_2,..., y_n)$ and $Z=(z_1, z_2,..., z_n)$.

For data from a sample, the Pearson correlation can be calculated as follows,

1 $$\rho _{{XY}} = \frac{{\sum\limits_{{i = 1}}^{n} {x_{i} y_{i} } - n\bar{x}\bar{y}}}{{(n - 1)s_{X} s_{Y} }}.$$

$s_X$, $s_Y$ are the sample standard deviations of *X* and *Y*.

Since the Pearson correlation is invariant under shifting and positive scaling of the two variables, this implies that $\rho _{{\tilde{X}}{\tilde{Y}}}$=$\rho _{XY}$ with ${\tilde{X}}=a(X-{\bar{x}})$ and ${\tilde{Y}}=b(Y-{\bar{y}})$ satisfying

2 $$\begin{aligned} \begin{array}{ccl} \sum _{i=1}^{n}{\tilde{x}}_i^2=\sum _{i=1}^{n}{\tilde{y}}_i^2=1\\ \sum _{i=1}^{n}{\tilde{x}}_i=\sum _{i=1}^{n}{\tilde{y}}_i=0 \end{array} \end{aligned}$$

where ${\bar{x}}$ and ${\bar{y}}$ are the sample means of *X* and *Y*. We can rewrite this as

3 $$\begin{aligned} \rho _{XY}=\rho _{{\tilde{X}}{\tilde{Y}}}=\sum _{i=1}^{n}{\tilde{x}}_i{\tilde{y}}_i. \end{aligned}$$

We assume that the samples are normalized to have zero mean and unit Euclidean norm.

**Fig. 5:**
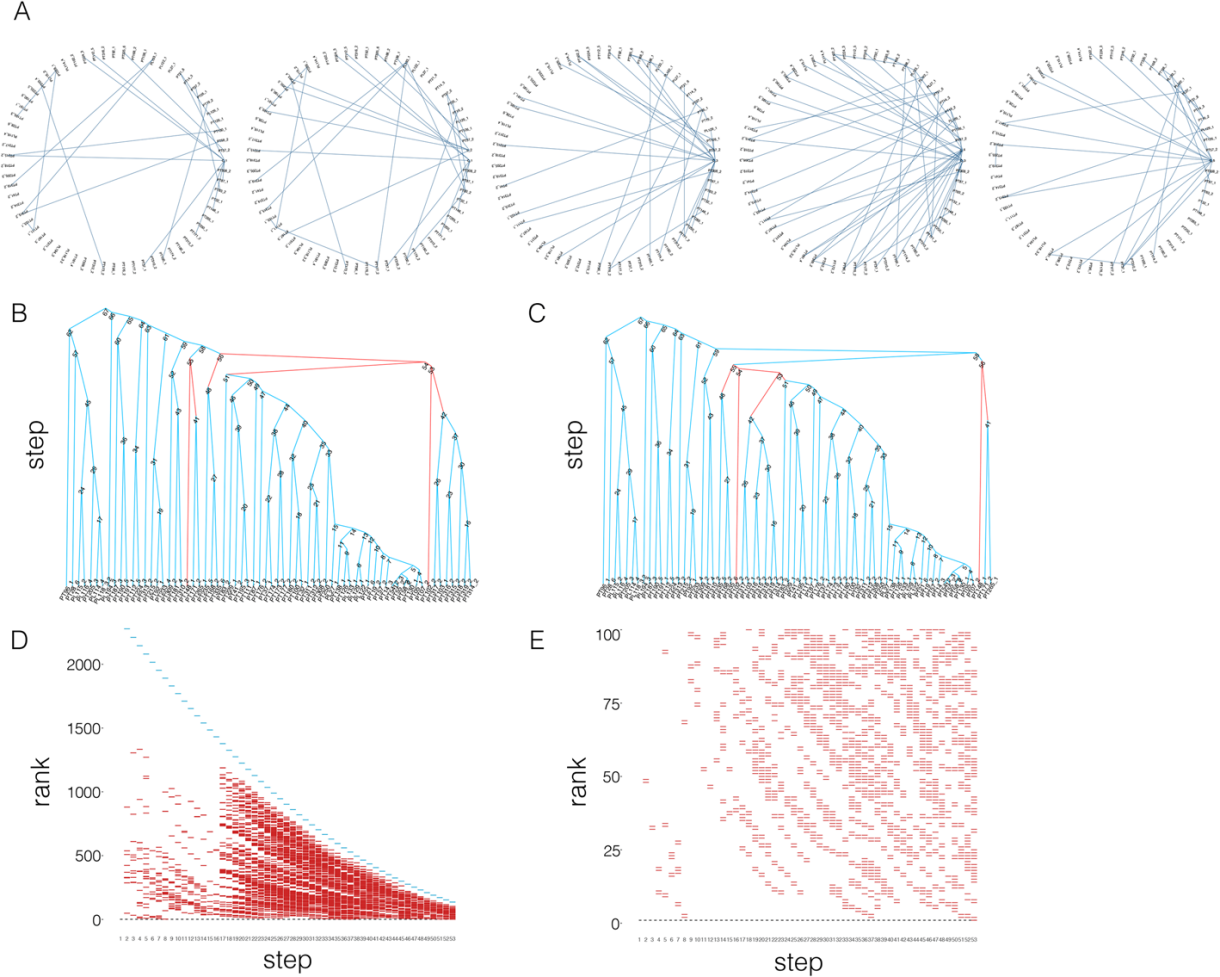
An example for average linkage hierarchical clustering with $d_a$ and $d_r$. **A**. The circle networks show the pairs where the distance is different in the ranks generated by $d_a$ and $d_r$ in step 2 to step 6. Nodes in network refer to samples for clustering. Edges refer to the distance where two samples are different in ranks in $d_a$ and $d_r$. $c_1$ refers to the class generated in step 1, $c_2$ refers to the class generated in step 2. **B**. Dendrogram for $d_a$. **C**. Dendrogram for $d_r$. The difference between the two dendrograms is colored in red. **D**. Distribution of ranks which are different in $d_a$ and $d_r$. E. Zoom in for the top 100 rank for D

Therefore, we have the modified Pearson distance

4 $$\begin{aligned} d_r (X,Y)=\root \of {1-|\rho _{XY}|}=\root \of {1-\left| \sum _{i=1}^{n}x_i y_i\right| }. \end{aligned}$$

By noting that $\sqrt{2-2\left| \sum _{i=1}^n x_i y_i\right| } = \textrm{min}(\Vert X-Y\Vert _2,\Vert X+Y\Vert _2)$, it suffices to prove

5 $$\begin{aligned} \textrm{min}(\Vert X-Y\Vert _2,\Vert X+Y\Vert _2) + \textrm{min}(\Vert Y-Z\Vert _2,\Vert Y+Z\Vert _2) \ge \textrm{min}(\Vert X-Z\Vert _2,\Vert X+Z\Vert _2). \end{aligned}$$

Since the Euclidean norm satisfies the triangle inequality, we have

6 $$\begin{aligned} \Vert X-Z\Vert _2= & {} \Vert X-Y + Y-Z\Vert _2&\le \Vert X-Y\Vert _2 + \Vert Y-Z\Vert _2 \end{aligned}$$

7 $$\begin{aligned} \Vert X-Z\Vert _2= & {} \Vert X+Y - (Y+Z)\Vert _2&\le \Vert X+Y\Vert _2 + \Vert Y+Z\Vert _2 \end{aligned}$$

8 $$\begin{aligned} \Vert X+Z\Vert _2= & {} \Vert X-Y + Y+Z\Vert _2&\le \Vert X-Y\Vert _2 + \Vert Y+Z\Vert _2 \end{aligned}$$

9 $$\begin{aligned} \Vert X+Z\Vert _2= & {} \Vert X+Y - (Y-Z)\Vert _2&\le \Vert X+Y\Vert _2 + \Vert Y-Z\Vert _2. \end{aligned}$$

It follows that

10 $$\begin{aligned} & {\text{min}}(\left\| {X - Z} \right\|_{2} ,\left\| {X + Z} \right\|_{2} ) \\ & \le {\text{min}}\left( {\left\| {X - Y} \right\|_{2} + \left\| {Y - Z} \right\|_{2} ,\left\| {X + Y} \right\|_{2} + \left\| {Y + Z} \right\|_{2} ,\left\| {X - Y} \right\|_{2} + \left\| {Y + Z} \right\|_{2} ,\left\| {X + Y} \right\|_{2} + \left\| {Y - Z} \right\|_{2} } \right) \\ & = {\text{min}}(\left\| {X - Y} \right\|_{2} ,\left\| {X + Y} \right\|_{2} ) + {\text{min}}(\left\| {Y - Z} \right\|_{2} ,\left\| {Y + Z} \right\|_{2} ). \\ \end{aligned}$$

It can be rewrited as,

11 $$\begin{aligned} \sqrt{2-2\left| \sum _{i=1}^n x_i z_i\right| } \le \sqrt{2-2\left| \sum _{i=1}^n x_i y_i\right| }+\sqrt{2-2\left| \sum _{i=1}^n y_i z_i\right| }, \end{aligned}$$

$$d_r(X,Z) \le d_r(X,Y)+d_r(Y,Z)$$ holds.

**Fig. 6:**
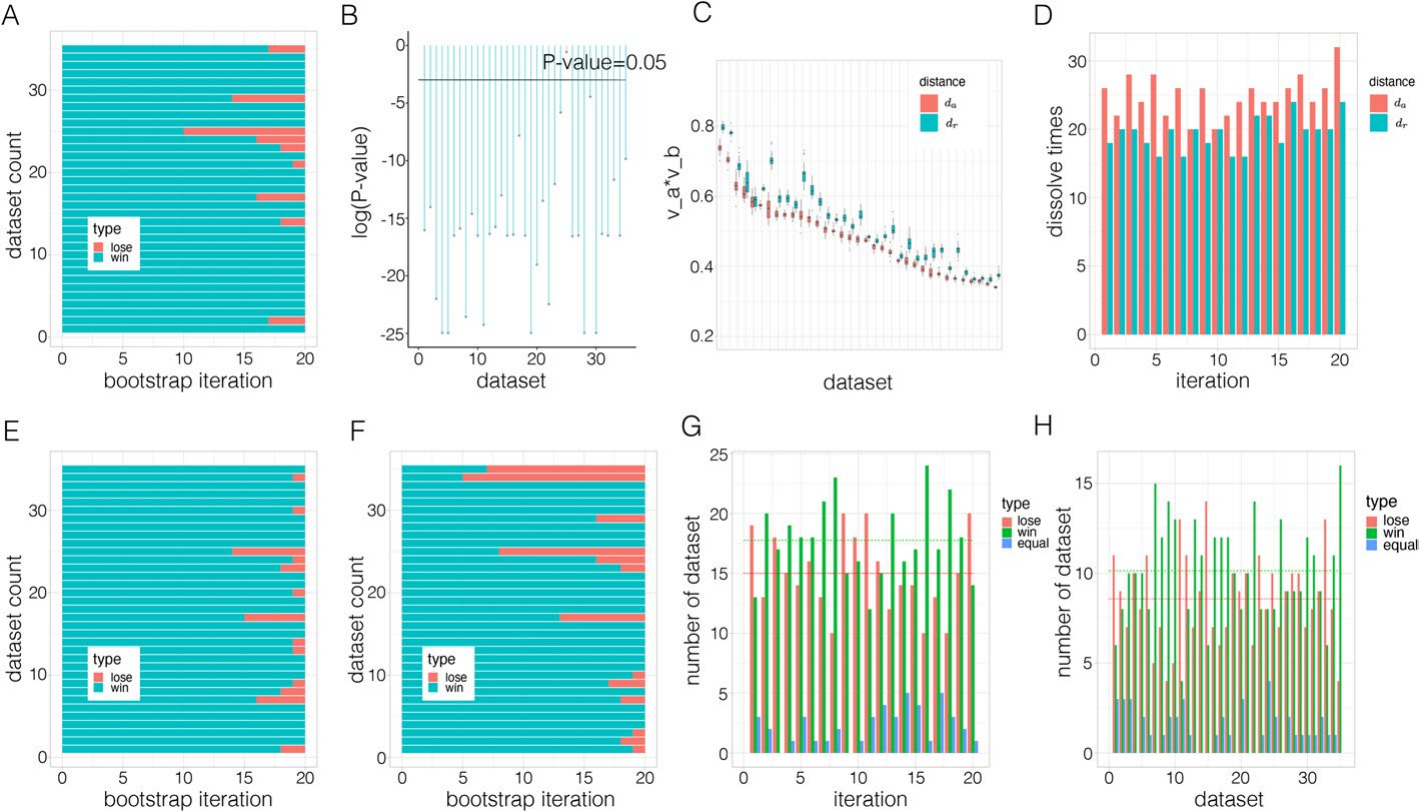
Result for robustness test on $d_a$ and $d_r$. **A**, **B**, **C**, **D** are results obtained using Pearson correlation as $\rho$ on PAM. **A**. The number of times $d_r$ win over 20 iterations in each dataset. Each row corresponds to one dataset. True difference in the count of win and lose event is not equal to zero (*P*-value=$3.41e-14$). **B**. *P*-values in testing the difference between the number of times imply $d_r$ wins in all 35 datasets. Each point corresponds to one dataset. **C**. Each box represents one $\upsilon$ value over 20 iterations per dataset. We compared the box plot for $d_a$ and $d_r$ in each dataset. The datasets in C have been reordered to fit the decrease of y value to show the trend more clearly. **D**. The number of classes “dissolved” in $d_a$ and $d_r$ across all 20 iterations. (*P*-value=0.005). **E**. Result for Spearman correlation as $\rho$ in PAM clustering. (*P*-value=$1.08e-13$). **F**. Result for uncentered Pearson correlation as $\rho$ in PAM clustering. True difference in the count of win and lose event is not equal to zero (*P*-value$=3.39e-13$).**G**, **H** are results for Pearson correlation as $\rho$ in hierarchical clustering, considering all internal nodes as classes. G. Result for comparing $d_a$ and $d_r$ by the number of times classes “dissolved” in 35 datasets over 20 iterations. The number of times $d_r$ wins, loses, or is equal to $d_a$. The green horizontal line represents the average number across all the iterations where $d_r$ wins. The red horizontal line represents the average number across all the iterations where $d_r$ loses. True difference in the count of win and lose events is not equal to zero (*P*-value=0.020).H. Result for comparing $d_a$ and $d_r$ per dataset. (*P*-value=0.019)

We prove the triangle inequality of $d_r$ when $\rho$ is Spearman correlation and uncentered Correlation in Additional file 1.

### Evaluation

We compared our modified absolute Pearson correlation distances $d_r$ to $d_a$ as well as $d_s$ through their performances in clustering real gene expression datasets. We used microarray data, which includes 16 gene time-series profiles [23] and 35 datasets for clustering of cancer samples [24], as well as seven single cell gene expression data [25–31] sets for clustering cells. As clustering algorithm, we used hierarchical clustering and PAM. Clustering is performed on both genes and samples. The input of a clustering task is a distance matrix and the output is a partitioning which gives the clusters.

For the sample clustering, we selected the number of clusters, *k*, according to the benchmark. We evaluated the clustering result by examining how consistent the clusters are with the benchmarks in terms of ARI [21]. A greater ARI value indicates higher concordance between our $d_r$-based partition and the benchmark partition.

**Fig. 7:**
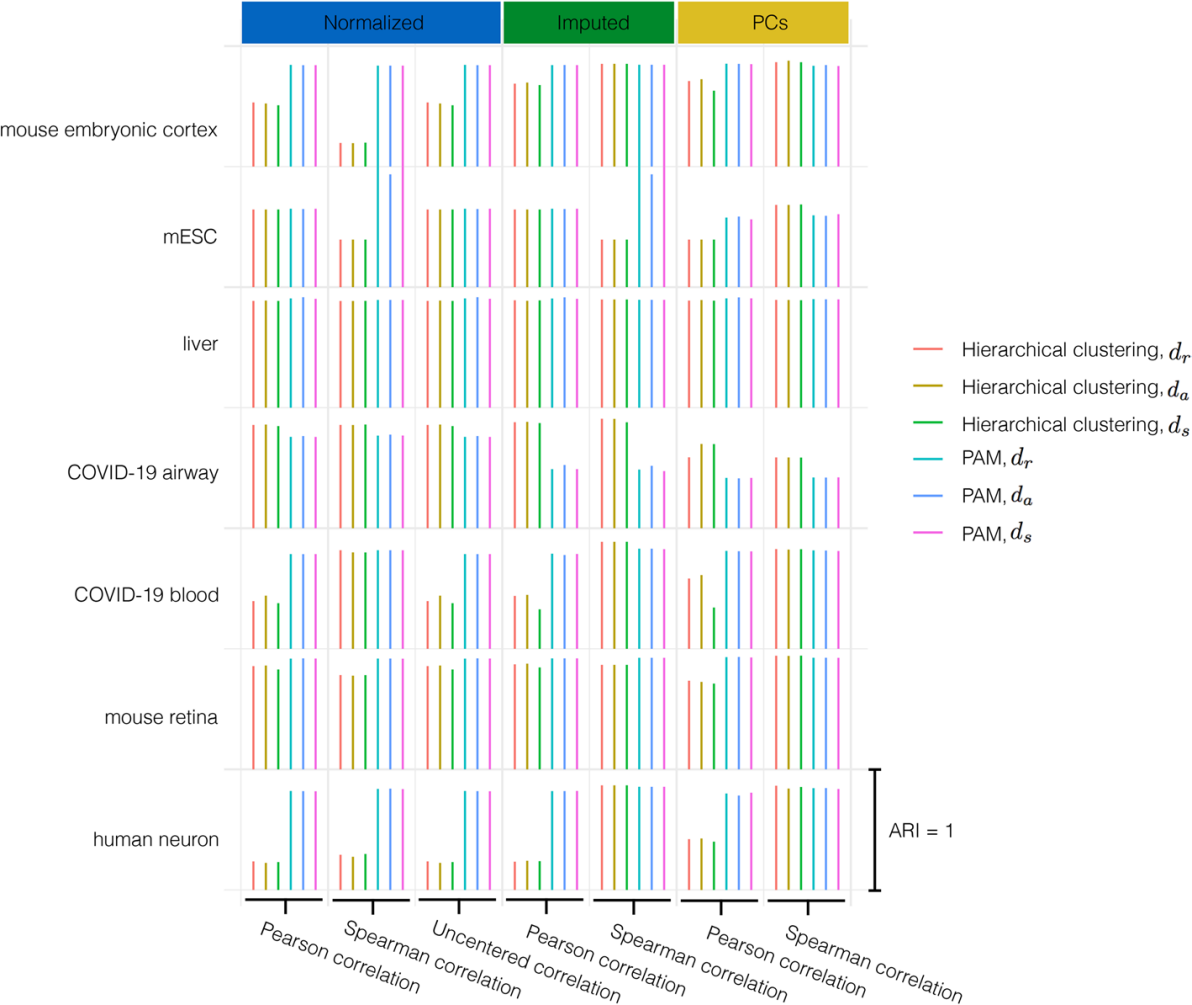
Performance in single cell clustering. The length of lines imply the value of ARI when apply $d_r$, $d_a$ and $d_s$ based distance to clustering single cell gene expression. The length of line which refers to ARI equals to 1 is shown at the bottom right. The experiments are conducted in seven single cell gene expression with data been normalized, imputed and PCs after dimension reduction

For the gene clustering, we evaluated clustering performance by gene functional analysis [19]. The number of clusters was determined according to Calinski-Harabasz Index ($CH_{index}$) [32] as follows. The $CH_{index}$ is given as

12 $$\begin{aligned} CH_{index}=\frac{SS_B}{SS_W} \times \frac{N-k}{k-1}, \end{aligned}$$

where *k* is the number of clusters, *N* is the total number of samples, $SS_W$ is the overall within-cluster variance, and $SS_B$ is the overall between-cluster variance. A higher $CH_{index}$ value implies a better solution. We used the value of *k* which corresponds to the peak or at least an abrupt elbow on the line-plot of $CH_{index}$ value.

After obtaining the clusters, we performed GO enrichment for each generated cluster with R package [33–35]. For each cluster generated by $d_a$, we got a set of significant GO terms with *P*-value < 0.05, denoted as $r_1$. Similarly, for the clusters generated by $d_r$, we got a set of significant GO terms $r_2$. After that, for the two result lists $r_1$ and $r_2$, we counted the number of times that the GO term of $r_1$ has smaller *P*-value than that of $r_2$, denoted as $\#(r_1< r_2)$, and the number of times that GO term of $r_2$ has smaller *P*-value than that of $r_1$, denoted as $\#(r_2<r_1)$. Then we calculated

13 $$\begin{aligned} comparison(r_1,r_2)=\log (\frac{\# (r_1< r_2)}{\# (r_2<r_1)}). \end{aligned}$$

Positive values of $comparison(r_1,r_2)$ imply that $r_1$ is better than $r_2$, and negative values imply the opposite. Therefore, the negative values mean $d_r$ wins $d_a$ in this dataset. If we change the order of the results under comparison ($r_1$,$r_2$) or ($r_2$,$r_1$), it will only change the sign of the result, but not its absolute value. Besides, we also applied the statistical test on the *P*-value of the same GO term acquired by $d_a$ and $d_r$ to see whether the difference in the comparison pair is significant.

### Robustness test

To test the robustness of clusters with different distance measures, we performed bootstrap experiments on the 35 microarray datasets for clustering cancer samples, and investigated the “dissolved” [22] event for the class given by different clustering processes. For each dataset, we first obtained an original partition $p_o$ from the original dataset. Then, for each dataset, suppose there are *n* samples in total, we bootstrapped 100 times. For each time, we randomly selected *n* samples with replacement from the original dataset, and performed clustering on the resampled data to get a resulting partition $p_i$. We compared $p_i$ with $p_o$. Denote $c_{oj}$ as class *j* in $p_o$, $c_{ik}$ as class *k* in $p_i$. For each *i*, we calculated the Jaccard similarity, $J_{ijk}$, between each $c_{oj}$ and all $c_{ik}$. Then we calculated $J_{ij}=\max (J_{ijk})$. After repeating 100 times, for each $c_{oj}$ in $p_o$, we obtained 100 similarity values, respectively denoted $J_{ij}$ for each of the bootstraps. If $J_{ij} < 0.5$, we take $c_{oj}$ as having “dissolved” in bootstrap *i*. We counted the number of times the class $c_{oj}$ dissolved in 100 bootstraps. If this frequency is larger than 40, we regard $c_{oj}$ as being dissolved in the experiment. We repeated the bootstrap process for multiple iterations. We tested the robustness of the class by comparing the times it dissolved in multiple iterations. Finally, we compared the performance of $d_a$ and $d_r$ by comparing the robustness of the classes they generated.

We also investigated sample pairs for robustness. We selected sample pairs that are clustered together to see whether they are consistently clustered together across multiple runs, in which case, the result for the sample pair is robust. Similarly, we examined sample pairs that are not clustered together to see if they are consistently placed in different classes. For each sample pair *i* and *j* in one dataset, we counted the number of times $n_1$ they are sampled together in 100 bootstraps, the number of times $n_2$ they are clustered in the same class, and the number of times $n_3$ they are clustered in different classes. If $n_2 >n_3$, then this pair is decided as consistently clustered, otherwise they are consistently not clustered together. For each sample pair (consistently clustered), we calculated the ratios $n_2/n_1$ as well as the median value $m_{together}$ for all non-zero ratio value. This is repeated for $n_3/n_1$ and their median, $m_{not\_together}$, for a sample pair consistently not clustered together. Then we calculated $\upsilon =m_{together}* m_{not\_together}$. A larger $\upsilon$ implies a more robust clustering. We recorded this as a “win” event for $d_r$ if $\upsilon _r > \upsilon _a$. For 35 files, we got a list of $\upsilon$ for $d_r$ and $d_a$. We did Wilcox test for the list of $\upsilon _r$ and $\upsilon _a$ with an alternative hypothesis as the true location shift is not equal to 0. To see whether $\upsilon$ for $d_r$ is significantly larger than $\upsilon$ for $d_a$.

## Results

### Comparison with squared correlation distance ($d_s$)

The squared correlation distance $d_s$ is analytically less sensitive than $d_r$ in responding to changes in $\rho$ (see Fig. 1). The behavior of $d_r$ is closer than $d_s$ to $d_a$, making it better as a replacement for $d_a$ in the tasks where $d_a$ performs well. This observation is confirmed by our empirical tests.

We compared the performance of $d_r$ and $d_s$ in clustering tasks, using 35 datasets from a previous work [24]. The samples in each dataset were assigned a label such as disease or healthy. We applied normalization to each dataset by scaling each gene to the standard normal distribution. We then performed hierarchical clustering and PAM, with the number of clusters *k* set as the number of unique labels in each dataset. We evaluated the performance by ARI [21], which measures the consistency between cluster partition and benchmark labels (see Additional file 1: Fig. S1). We applied the statistical test on ARI corresponding to cluster partition for 35 datasets with $d_r$-based clustering and $d_s$-based clustering. We compared the pairwise ARI values of $d_r$ and $d_s$ in the experiments with hierarchical clustering. The result demonstrated that the ARI values of $d_r$ are significantly higher than that of $d_s$ (*P*-value $< 0.1$ on Wilcoxon test) when the paired ARI values are different (see Fig. 2). That means $d_r$-based clustering outperformed $d_s$, especially when the clustering method is hierarchical clustering and $\rho$ is Pearson correlation and uncentered Pearson correlation in the sample clustering test.

In Additional file 1: Fig. S2, we show comparison results between $d_r$ and $d_s$ in gene clustering experiments using hierarchical clustering and PAM, with $\rho$ set to Pearson correlation, Spearman correlation, and uncentered Pearson correlation. As data, we used 16 time-series profile datasets with normalization from a previous work [23]. For each dataset, we calculated the distances $d_r$ and $d_a$ for pairwise gene profiles, resulting in the distance matrices $M_r$ and $M_a$. Then, we applied hierarchical clustering and PAM for each distance matrix, estimating the number of clusters *k* by $CH_{index}$. Since the datasets do not have a reference partition for genes, we evaluated the performance with biological functional analysis [19]. The clustering result with higher scored GO terms is considered as the better solution. See Fig. 3, we extracted comparison pairs with significant difference in *P*-value (for the corresponding GO-terms generated by $d_r$ and $d_s$). The statistical test also shows the win event of $d_r$ is significantly more than that of $d_s$ (*P*-value$<0.05$, Wilcoxon test). It implies that $d_r$ drives to better performance in gene cluster than that of $d_s$.

### Comparison with absolute correlation distance ($d_a$)

We first evaluated the performance of $d_r=\sqrt{1-|\rho |}$ and the $d_a=1-|\rho |$ on sample clustering. For the hierarchical clustering, complete and single linkage resulted in identical dendrograms. When $\rho$ is Pearson correlation, the hierarchical clustering in average linkage using both $d_a$ and $d_r$ resulted in similar ARI across all 31 datasets (see Additional file 1 : Fig. S3). When $\rho$ is Spearman correlation or uncentered Pearson correlation, both $d_a$ and $d_r$ resulted in the same ARI in at least 27 datasets, for both methods of clustering. For those datasets with different ARI in comparison, the number of times $d_r$ outperforms $d_a$ is close to the number of times $d_a$ outperforms $d_r$. As an example, in PAM, $d_r$ outperformed $d_a$ 5 times while $d_a$ outperformed $d_r$ 3 times when $\rho$ is Spearman correlation. These results show that they have comparably good performance in our sample clustering experiment. The limited samples in datasets may induce the minor difference in the clustering result.

We then evaluated the performance of $d_r$ and the $d_a$ on gene clustering. For hierarchical clustering with average linkage, $d_r$ outperformed $d_a$ in 10, 9, and 10 datasets among 16 datasets when $\rho$ is any of Pearson correlation, Spearman correlation, and Cosine similarity respectively (see Additional file 1 : Fig. S4). In PAM experiments, $d_r$ outperformed $d_a$ in 9, 12, 7. The two measures outperformed each other nearly equal number of times. If we only compare pairs with significant difference in *P*-value for the corresponding GO-terms generated by $d_r$ and $d_a$, see Fig. 4, the statistical test shows the win events of $d_r$ are more than that of $d_a$ (*P*-value$<0.1$, Wilcoxon test), if exclude uncentered Pearson correlation with PAM.

Besides comparing $d_r$, $d_a$, $d_s$ with correlations, we also made the comparison between different types of correlation measures when the form of distance formula is the same. See Additional file 1: Fig. S5 and Fig. S6, the performance of Pearson correlation and uncentered Pearson correlation are more similar compared to Spearman correlation. Moreover, when the clustering method is hierarchical clustering, Spearman correlation outperforms the other two in more datasets, while this observation is not found when the clustering method is PAM.

### Detailed analysis with an example

To see how $d_r$ and $d_a$ lead to different clustering results, we used one dataset as an example and observed the clustering process. We used the 18-th dataset [36] in the sample clustering experiment, performing hierarchical clustering, using Pearson correlation as $\rho$.

In the beginning, the distance matrices $M_r$ and $M_a$ calculated according to $d_r$ and $d_a$ are the same in rank, in the sense that if we sort the values in $M_r$ and $M_a$ increasingly, the two lists will have the same order. To perform hierarchical clustering, for each step, we updated the distance matrix, find the closest pair of clusters, and merge them into one cluster. We repeated the step until all items are clustered into a single cluster. For hierarchical clustering with complete linkage and single linkage, $d_r$ and $d_a$ lead to the same resultant dendrogram because they only take maximum or minimum distance values when calculating the distance between clusters, thus introducing no new value of distance during the entire clustering process. For hierarchical clustering with average linkage, the same two samples are merged at the first step. Since an average distance is computed for the newly generated cluster, a difference in rank emerges. In Fig. 5A, the circle network shows the pairs which are different in the ranks of the distance sets generated by $d_a$ and $d_r$ in Steps 2 to 6. In this dataset, $d_a$ and $d_r$ led to the same ARI even though the resultant dendrograms are different in structure. The dendrogram for $d_a$ is shown in Fig. 5B and that for $d_r$ in Fig. 5C. The difference between the two dendrograms is colored in red. Figure 5D shows the distribution of the ranks which are different.

From step 2 to step 52, there exist different ranks in the distance of pairs in two distance experiments. However, the pairs of rank 1 are the same, showing that both $d_a$ and $d_r$ led to the same two clusters being merged into a new cluster. In step 53, the pair of rank 1 starts to differ, showing that different clusters in two distance experiments have been selected. This difference is reflected in the resultant dendrogram. As shown in Fig. 5B and C, for $d_a$, $c_{42}$ and “$PT102\_2$” have been merged, while for $d_r$, $c_{42}$ and $c_{51}$ have been merged (*c* represents the internal node in the dendrogram).

In the sample clustering experiment, due to the scarcity in the number of pairs (the maximum number of samples in a single dataset is 248 among datasets in this sample clustering experiment), the difference in ARI only occurred in 4 out of 35 datasets. In the gene clustering experiment, the boosted number of pairs enlarged the differences in the dendrogram, hence the partition is different in all 16 time-series datasets.

### Robustness test

We compared the methods’ robustness with bootstrap experiments in clustering cancer samples on 35 microarray datasets. This is done by examining the number of sample pairs that are consistently clustered across 20 iterations. In each iteration, we resampled 100 times for each dataset. For PAM, $d_r$ displayed more robust clustering than $d_a$. Figure 6A, B, C and D are for comparing $d_r$ and $d_a$ through PAM clustering using Pearson correlation as $\rho$. Figure 6A shows the number of times $d_r$ achieved a win over 20 iterations in each dataset. $d_r$ achieved more wins in 34 datasets among 35 datasets (see Fig. 6B). Figure 6C shows the box plot for $\upsilon$ over 20 iterations and 35 datasets.

Figure 6D shows the results where we evaluated the robustness through the number of times a class is “dissolved”. The number of classes dissolved through $d_a$ is larger than it in $d_r$ in all 20 iterations. Hence, $d_r$ led to more robust clustering results, consistent with our earlier results in Fig. 6A, B, C. Similar results are obtained when $\rho$ is Spearman correlation and Cosine similarity, as shown in Fig. 6E and F.

For the hierarchical clustering, we examined all the internal nodes for the number of times that the classes dissolved for each dataset. Figure 6G shows the number of datasets where $d_r$ achieved a win. Figure 6H shows the comparison according to each dataset. Both figures show that $d_r$ achieved a win more times than $d_a$. Across 20 iterations, the average number of times when $d_r$ wins is larger than that when $d_a$ wins. We also applied the statistical test to win event count in both sample-pair experiments and dissolved experiments. The results show the use of $d_r$ resulted in significantly more robust clustering than $d_a$ in both hierarchical and PAM clustering.

### Clustering in single cell gene expression

We further examined the performance of clustering based on $d_r$, $d_a$, and $d_s$ in seven single cell gene expression datasets, including human neuron [25], mouse retina [26], blood in COVID-19 patients and healthy controls [27], airway in COVID-19 patients and healthy controls [28], human liver [29], mESC [30] and mouse embryonic cortex [31]. The clustering benchmark is downloaded from the corresponding paper, which is the cell type assigned by the previous study with annotation according to marker genes, while the mESC’s cluster partition is the cell cycle the cell belongs to. For each single cell data set, we first filtered out samples and genes according to zero numbers, expression average, and variance. Then three kinds of data preprocessing are conducted, normalization, imputation by scImpute [37], and dimension reduction by principal component analysis (PCA), then the top 50 PCs are used for clustering. We applied $d_r$, $d_a$, and $d_s$ based distance to do clustering in the three format single cell datasets. The result shows imputation and dimension reduction can improve the clustering performance in single cell data. PAM outperforms hierarchical clustering in more data sets. Moreover, if the experiment setup, such as data preprocessing, clustering method, and correlation type is the same, then the performances of $d_r$, $d_a$, and $d_s$ only have a slight difference (see Fig 7). That makes $d_r$ a promising replacement to $d_a$. Given $d_r$ satisfies the triangle inequality, it provides us more possibility to faster data localization and speeds up clustering.

## Discussion and conclusion

The absolute correlation distance $d_a=1-|\rho |$ is widely used in biological data clustering despite its shortcoming of not satisfying the triangle inequality. More recently, a metric distance $d_s$ was proposed. In this paper, we proposed an alternative metric, $d_r$. We first show $d_r$ to be better than $d_s$, both analytically as well as in empirical tests at biological clustering. In our tests on gene clustering using 16 normalized time-series datasets, sample clustering in 35 expression datasets, cell clustering in seven single cell datasets, both $d_r$ and $d_a$ have comparable performances in both gene clustering and sample clustering, using both hierarchical as well as PAM clustering. However, $d_r$-based clustering led to more robust results. The robustness of $d_r$-based clustering is also supported by evaluation based on the number of times that a class “dissolved”. That makes $d_r$ a good option when measuring correlation-based distances, which have comparable accuracy, higher robustness. Moreover, $d_r$ can be applied in accelerated clustering which makes use of the triangle inequality [13].

$d_r$ is one special case of $\sqrt{1-(|\rho |)^{\alpha }}$ when $\alpha =1$. When $\alpha$ is set as other values (see Additional file 1 : Fig. S7), the performance of sample clustering is shown in Additional file 1: Fig. S8, while the equation may also fulfill the triangle inequality, which requires further discussion.

## Supplementary Information

**Additional file 1: ** The proof of $d_r$ fulfilling the triangle inequality for Spearman correlation or uncentered Pearson correlation as $\rho$ and additional analysis are provided.

## Data Availability

Data for evaluation are collected from published papers. The 16 gene time-series profiles are downloaded from [23] and 35 microarray datasets for clustering of cancer samples are downloaded from [24]. Seven single cell gene expression profiles and corresponding cluster partition can be found in previous studies [25–31]. Code and material are available at https://github.com/jiaxchen2-c/clustering$\_$evaluation$\_$triangular$\_$inquality.

## Abbreviations

$$d_a=$$: $$1-|\rho |$$
$$d_r=$$: $$\sqrt{1-|\rho |}$$
$$d_s=$$: $$\sqrt{1-\rho ^2}$$
PAM: Partitioning around medoids
ARI: Adjusted Rand index
GO-term: Gene Ontology term
$CH_{index}$: Calinski-Harabasz Index
PCA: Principal component analysis
