# Correction: On triangle inequalities of correlation-based distances for gene expression profiles

**DOI:** 10.1186/s12859-023-05358-1

**Published:** 2023-05-31

**Authors:** Jiaxing Chen, Yen Kaow Ng, Lu Lin, Xianglilan Zhang, Shuaicheng Li

**Affiliations:** 1 Department of Computer Science, City University of Hong Kong, Hong Kong, China; 2 Department of Computer Science, Beijing Normal University - Hong Kong Baptist University United International College, Zhuhai, People’s Republic of China; 3 State Key Laboratory of Pathogen and Biosecurity, Beijing Institute of Microbiology and Epidemiology, Beijing, 100071 People’s Republic of China

**Correction: BMC Bioinformatics (2023) 24:40** 10.1186/s12859-023-05161-y

Following publication of the original article [1], it was reported that the article entitled “On triangle inequalities of correlation-based distances for gene expression profiles” was published in the regular issue of this journal instead of in the supplement issue.

The details of the supplement in which this article ought to have been published are given below:

**About this supplement**

This article has been published as part of BMC Bioinformatics Volume 23 Supplement 3, 2022: Selected articles from the International Conference on Intelligent Biology and Medicine (ICIBM 2021): bioinformatics. The full contents of the supplement are available online at https://bmcbioinformatics.biomedcentral.com/articles/supplements/volume-23-supplement-3.

The publisher apologizes for any inconvenience caused.

