# Supplementary material for: On triangle inequalities of correlation-based distances for gene expression profiles
Source: BMC Bioinformatics. 2023 Feb 8;24:40. doi: 10.1186/s12859-023-05161-y (PMC9906874; doi:10.1186/s12859-023-05161-y)
Supplement: Supplementary file 1 — Additional file 1: The proof of $d_r$dr fulfilling the triangle inequality for Spearman correlation or uncentered Pearson correlation as $\rho$ρ and additional analysis are provided. [file 12859_2023_5161_MOESM1_ESM.pdf]

# Supplementary Note

## Proof of $d_r$ fulfilling the triangle inequality for Spearman correlation as $\rho$

The case of $d_r(X,Y) = \sqrt{1-|\rho(X,Y)|}$, when $\rho$ is the Spearman correlation, can be regarded as a special case of $d_r(X,Y) = \sqrt{1-|\rho(X,Y)|}$, when $\rho$ is Pearson correlation, where X=$(x_1, x_2, ..., x_n)$, Y=$(y_1, y_2, ..., y_n)$ and $x_1, x_2, ..., x_n$, $y_1, y_2, ..., y_n$ are integers. Since the inequality holds for the case of Pearson correlation, it trivially holds here.

## Proof of $d_r$ fulfilling the triangle inequality for uncentered Pearson correlation as $\rho$

We define the distance of $X$ and $Y$ by $d_r(X,Y) = \sqrt{1-|\rho_{XY}|}$, where $\rho$ is the uncentered Pearson correlation. Let $X = (x_1, x_2, ..., x_n)$, $Y = (y_1, y_2, ..., y_n)$ and $Z = (z_1, z_2, ..., z_n)$.

For any data from a sample, the uncentered Pearson correlation can be calculated as follows

$$\rho_{XY} = \frac{1}{n}\sum_{i=1}^{n}(\frac{x_i}{\sigma_x^{(o)}})(\frac{y_i}{\sigma_y^{(o)}}) \tag{1}$$

where $\sigma_x^{(o)} = \sqrt{\frac{1}{n}\sum_{i=1}^{n} x_i^2}$, $\sigma_y^{(o)} = \sqrt{\frac{1}{n}\sum_{i=1}^{n} y_i^2}$.

$\rho_{XY}$ can be written as cosine similarity,

$$cos\theta = \frac{X \cdot Y}{|X||Y|} = \frac{\sum_{i=1}^{n} x_i y_i}{\sqrt{\sum_{i=1}^{n} x_i^2}\sqrt{\sum_{i=1}^{n} y_i^2}}, \tag{2}$$

where $\theta$ is the angle between $X$ and $Y$.

Since the cosine similarity is invariant under separate changes in scale in the two variables, this implies that $\rho_{\tilde{X}\tilde{Y}}$=$\rho_{XY}$ with $\tilde{X} = a(X)$ and $\tilde{Y} = b(Y)$ satisfying

$$\textstyle\sum_{i=1}^{n} \tilde{x}_i^2 = \sum_{i=1}^{n} \tilde{y}_i^2 = 1. \tag{3}$$

We can rewrite this as

$$\rho_{XY} = \rho_{\tilde{X}\tilde{Y}} = \sum_{i=1}^{n} \tilde{x}_i \tilde{y}_i. \tag{4}$$

Without loss of generality, we assume that the samples are normalized to have zero mean and unit Euclidean norm. Therefore, we have modified uncentered Pearson correlation distance

$$d_r(X,Y) = \sqrt{1-|\rho_{XY}|} = \sqrt{1-\left|\sum_{i=1}^{n} x_i y_i\right|}. \tag{5}$$

To prove the triangle inequality of $d_r$, we need to prove

$$\sqrt{1-\left|\sum_{i=1}^{n} x_i y_i\right|} + \sqrt{1-\left|\sum_{i=1}^{n} y_i z_i\right|} - \sqrt{1-\left|\sum_{i=1}^{n} x_i z_i\right|} > 0, \tag{6}$$

which is equivalent to the triangle inequality of following Equation,

$$d_r(X,Y) = \sqrt{1-|\rho_{XY}|} = \sqrt{1-\left|\sum_{i=1}^{n} x_i y_i\right|}. \tag{7}$$

Since we have proved the triangle inequality for Equation 7 in the main article, it holds here too.

# Supplementary Figures

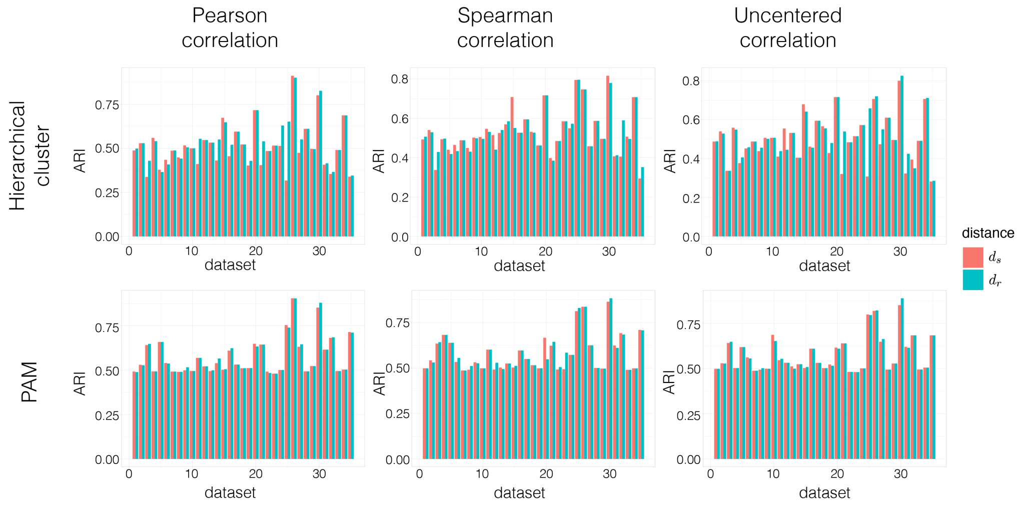


**Figure S1. Result of comparing $d_r$ and $d_s$ in sample clustering.** For each subfigure, the dataset is given on the $x$-axis, while the corresponding ARI value is on the $y$-axis. A larger ARI implies a better partitioning.

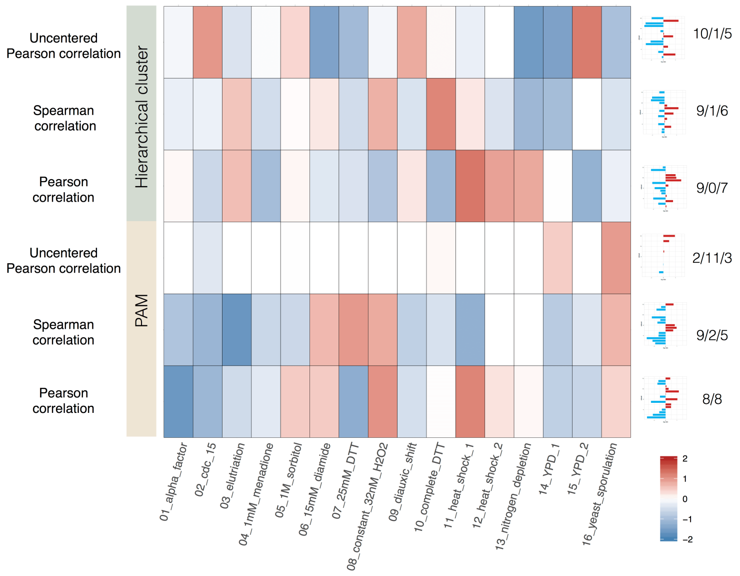


**Figure S2. Result of comparing $d_r$ and $d_s$ in gene clustering.** Each column corresponds to one time-series profile dataset. Each row corresponds to one comparison between $d_r$ and $d_s$. $\rho$ spans over different correlation measures used in the clustering. Color refers to the value of $comparison(r_1, r_2)$. Negative value implies that $d_r$ is better than $d_s$, and positive values implies the opposite. For each comparison combination, there is a barplot to the right of the corresponding row. The $x$-axis of the barplot refers to $comparison(r_1, r_2)$. The number at the right refers to the number of times $d_r$ wins, equal, and $d_s$ wins.

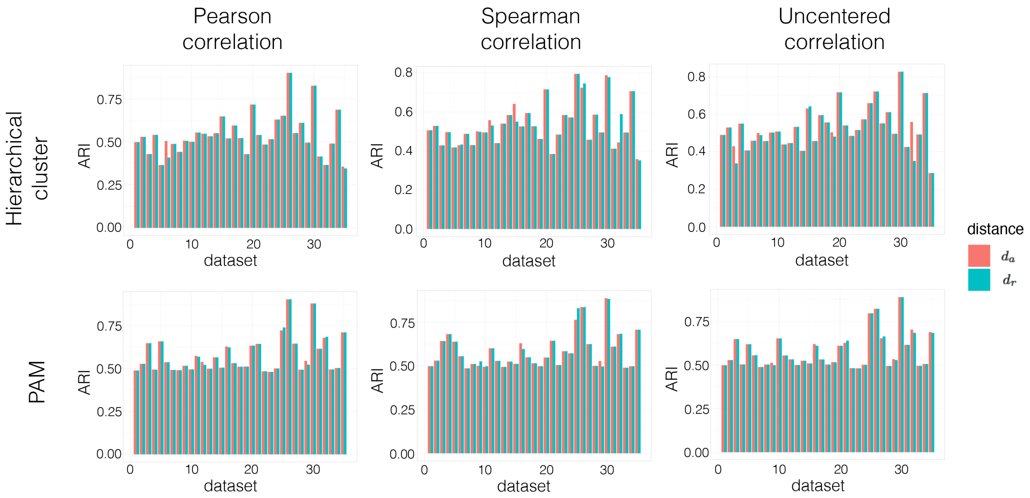


**Figure S3. Result of comparing $d_r$ and $d_a$ in sample clustering.** For each subfigure, different datasets are plotted along the $x$-axis, while ARI values are given on the $y$-axis. A larger ARI implies a better partitioning.

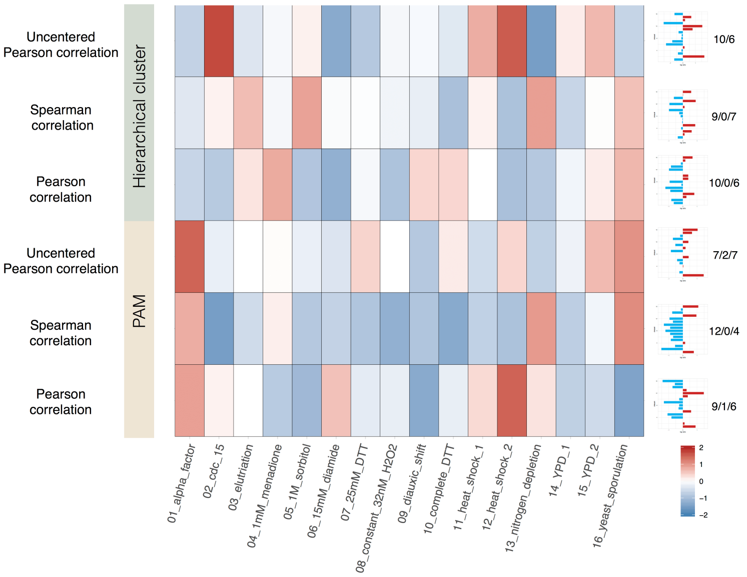


**Figure S4. Result of comparing $d_r$ and $d_a$ in gene clustering.** Each column corresponds to one time-series profile dataset. Each row corresponds to one comparison between $d_r$ and $d_a$ while $\rho$ is different correlation in certain clustering method. Color refers to the value of $comparison(r_1, r_2)$. Negative value implies that $d_r$ is better than $d_a$, and positive value implies the opposite. For each comparison combination, there is a barplot to the right of the corresponding row. The $x$-axis of the barplot specifies the comparison type $comparison(r_1, r_2)$. The number at the right gives the number of times $d_r$ wins, equal, and $d_a$ wins.

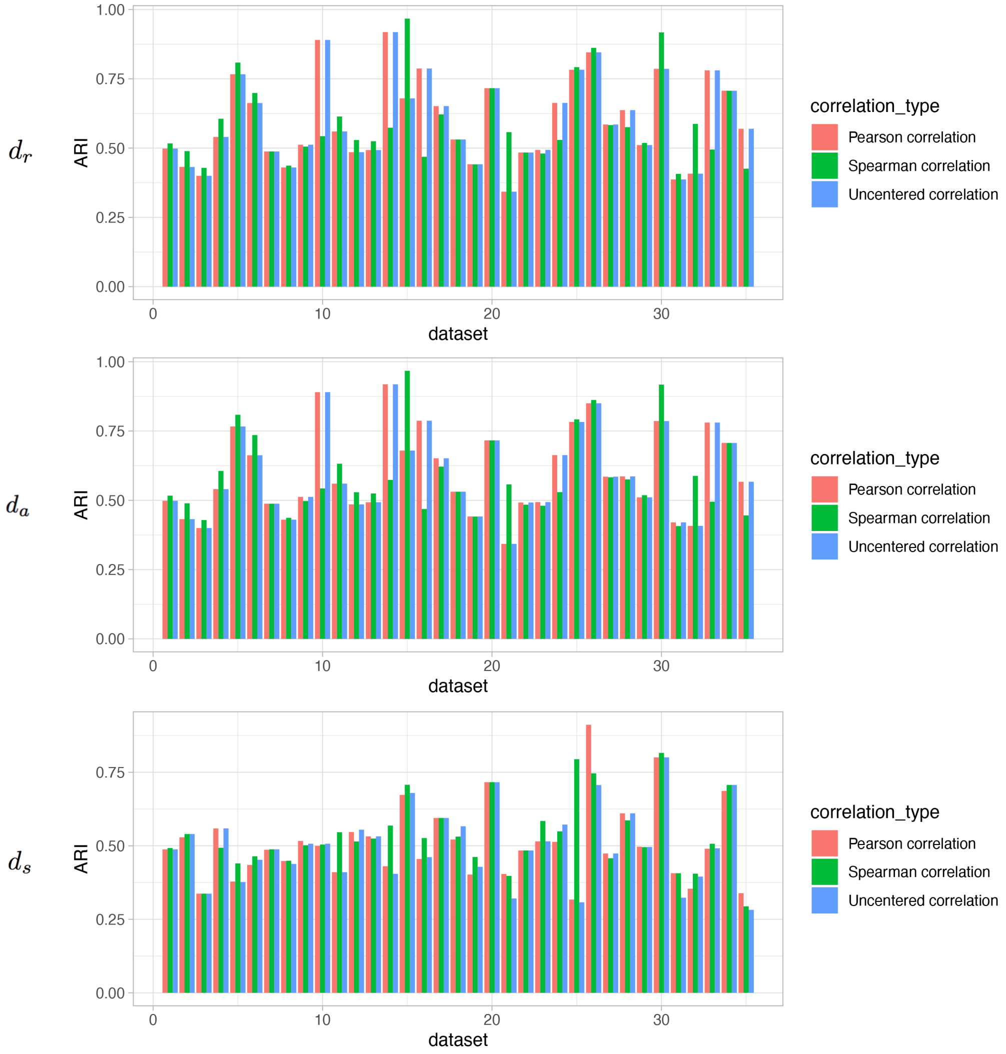


**Figure S5. Result of comparing correlation measures with the same form of distance formula, when the clustering method is hierarchical clustering.** For each subfigure, $y$-axis refers to ARI values in the sample clustering. Different datasets are plotted along the $x$-axis. Different colors refer to different correlation measures.

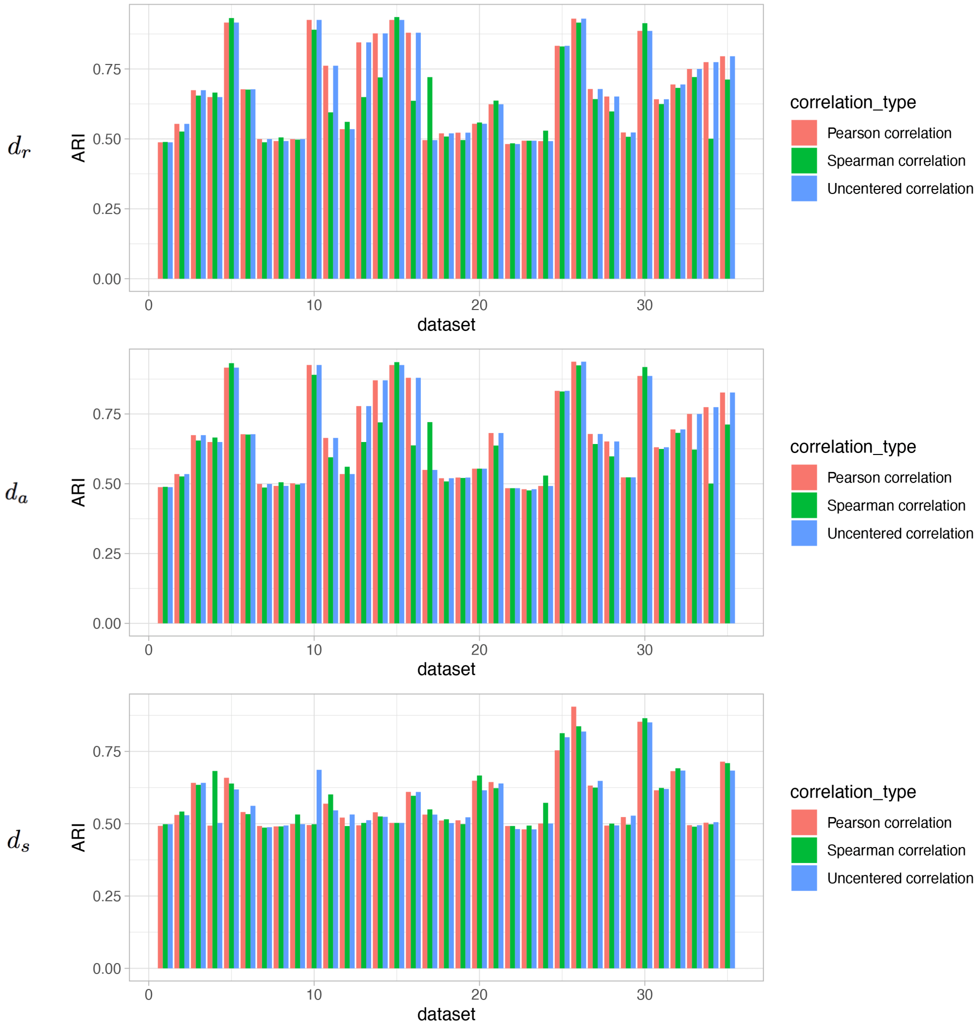


**Figure S6. Result of comparing correlation measures with the same form of distance formula, when the clustering method is PAM.** For each subfigure, $y$-axis refers to ARI values in the sample clustering. Different datasets are plotted along the $x$-axis. Different colors refer to different correlation measures.

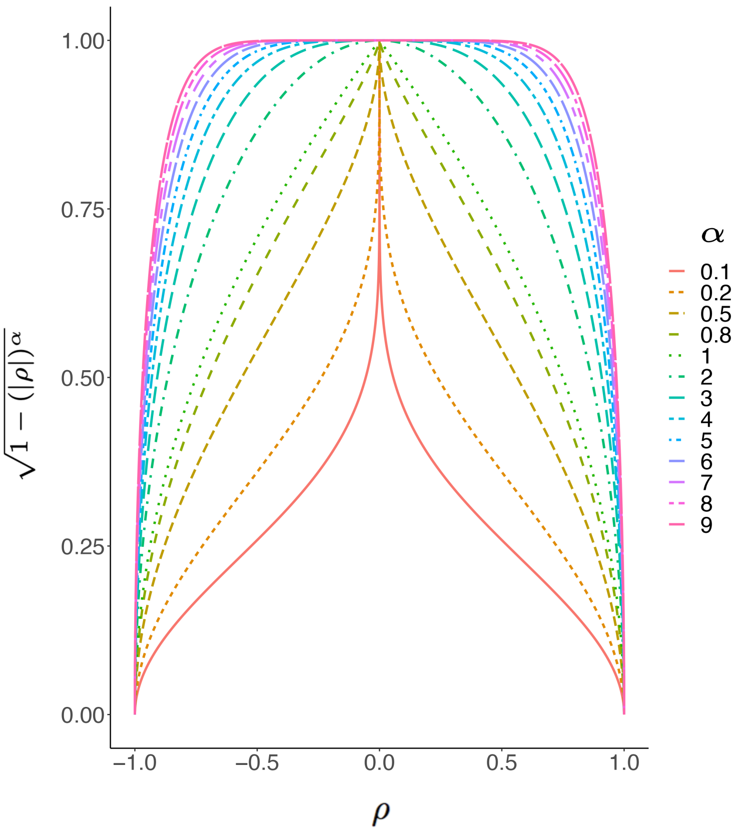


**Figure S7. Curve plot for $\sqrt{1-|\rho|^{\alpha}}$ when $\alpha$ is different.** $x$-axis is $\rho$ and $y$-axis refers to the corresponding value of the function.

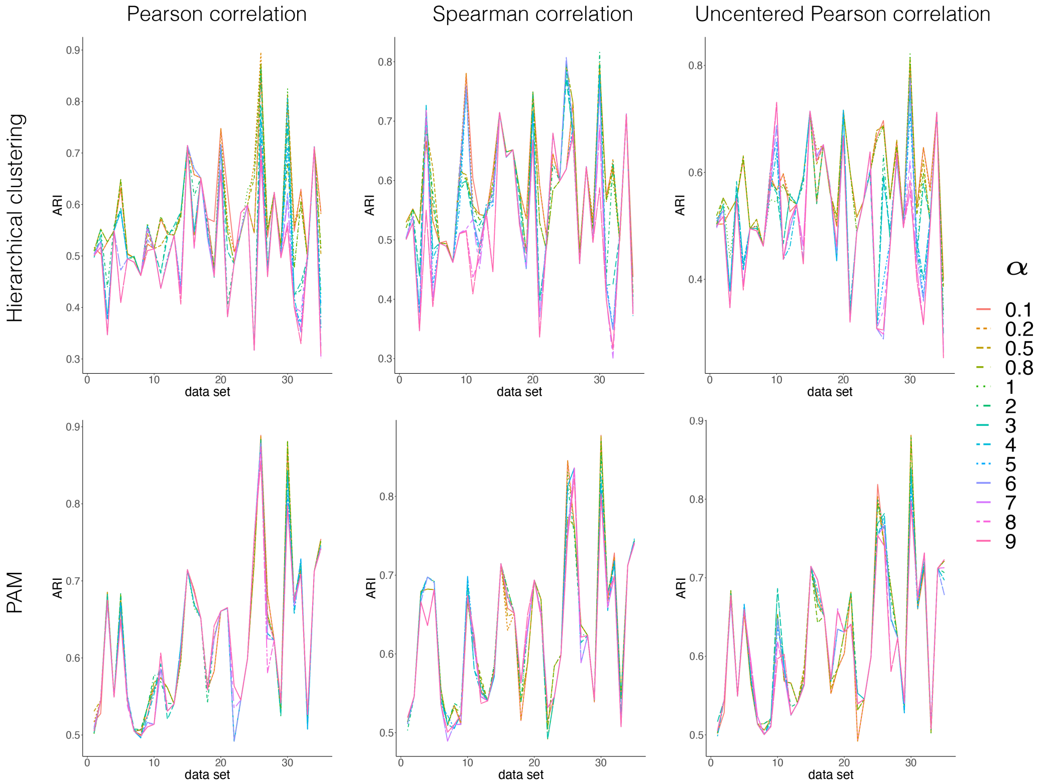


**Figure S8. Result of sample clustering for $\sqrt{1-|\rho|^{\alpha}}$ with different $\alpha$.** For each subfigure, the dataset is given on the $x$-axis, while the corresponding ARI value are on the $y$-axis. Six subfigures refer to experiments when $\rho$ is Pearson correlation, Spearman correlation and uncentered Pearson correlation with hierarchical clustering and PAM, respectively.
